# Bone marrow mesenchymal stem cell-derived Wnt5a inhibits leukemia cell progression *in vitro* via activation of the non-canonical Wnt signaling pathway

**DOI:** 10.3892/ol.2014.2117

**Published:** 2014-05-07

**Authors:** YA LI SHEN, QING LUO, YU XIA GUO, GAI HUAI ZHENG, JIE YU, YOU HUA XU

**Affiliations:** 1 Ministry of Education Key Laboratory of Child Development and Disorders, Key Laboratory of Pediatrics in Chongqing, Chongqing International Science and Technology Cooperation Center for Child Development and Disorders, Chongqing 400014, P.R. China; 2 Department of Hematology and Oncology, Children’s Hospital of Chongqing Medical University, Chongqing 400014, P.R. China

**Keywords:** bone marrow mesenchymal stem cell, HL60 cell line, leukemia, Wnt5a, Wnt signaling pathway

## Abstract

Leukemia is one of the most common malignancies in humans worldwide; however, the molecular mechanism of the effect of bone marrow mesenchymal stem cells (bMSCs) on leukemia cell growth remains unclear. The present study demonstrated that Wnt5a protein expression was significantly induced in bMSCs via an adenovirus vector (P<0.01). The results showed that the proliferation of HL60 cells, a leukemia cell line, was significantly inhibited when the cells were stimulated with the culture supernatant of adeno-Wnt5a bMSCs compared with the culture supernatants of bMSCs and adeno-vector bMSCs for 24 or 48 h (P<0.01). The promoted maturation levels of HL60 cells were also observed following stimulation with the culture supernatant of adeno-Wnt5a bMSCs (P<0.01). However, no significant difference was identified in the proliferation and maturation of HL60 cells among the three groups stimulated with the culture supernatants containing a neutralization antibody against Wnt5a. Furthermore, the bMSC-derived Wnt5a was found to influence the maturation and proliferation of the HL60 cells by enhancing the non-canonical Wnt signaling pathway, while inhibiting the canonical Wnt signaling pathway by upregulating the expression of receptor tyrosine kinase-like orphan receptor 2 and calcium/calmodulin-dependent protein kinase II, and suppressing the expression of β-catenin and cyclin D1. In conclusion, bMSC-derived Wnt5a modifies the proliferation and maturation of HL60 cells via activation of the non-canonical Wnt signaling pathway.

## Introduction

Leukemia is a multistep process involving the alteration of different pathways, which ultimately affect cell proliferation and maturation (1). However, the hematopoietic microenvironment of the bone marrow is also important in the development of leukemia. Bone marrow mesenchymal stem cells (bMSCs) are important elements of the hematopoietic microenvironment that frequently influence the development of leukemia via the secretion of hematopoietic growth factors (2). The communication between bMSCs and hematopoietic cells alters leukemia progression (3). A large number of proteins are involved in these regulatory pathways between bMSCs and hematopoietic cells, including Wnt5a, based on extracellular receptor-ligand interactions. Wnt5a is one of the most extensively studied proteins of the Wnt family and has a number of important functions in different types of cancer by antagonizing the canonical and inducing the non-canonical Wnt signaling pathways (4–6). Wnt5a also promotes the expansion and self-renewal of apoptosis-resistant transgenic hematopoietic stem cells (HSCs), as well as the self-renewal of leukemia cells *in vitro* (7,8). Our previous study showed that Wnt5a-overexpressing bMSCs regulate the maturation and proliferation of HL60 cells when the bMSCs are cocultured with HL60 cells (9). However, the molecular mechanisms of this process remain unclear.

Wnt signaling is important in embryonic development, adult homeostasis and tumor progression via the canonical and non-canonical β-catenin pathways. In addition, Wnt signaling is also responsible for primary acute myeloid and chronic lymphocytic leukemia (10). Receptor tyrosine kinase-like orphan receptor 2 (Ror2) is the coreceptor of Wnt5a and belongs to the Ror subfamily of cell surface receptors. Ror2 induces the activation of the non-canonical signaling pathway by binding to Wnt5a, which triggers the downstream signaling cascades, including Ca^2+^/calmodulin-dependent protein kinase II (CaMKII) (11). Therefore, we hypothesized that bMSC-derived Wnt5a influences the proliferation and maturation of HL60 cells via activation of the non-canonical Wnt signaling pathway.

The present study investigates whether bMSCs-induced Wnt5a was capable of regulating the matuation and proliferation of HL60 cells through stimulation with with culture supernatants containing Wnt5a protein obtained from bMSCs infected with adeno-Wnt5a, adeno-vector or normal bMSCs, and examines which Wnt signaling pathways are responsible for regulation.

## Materials and methods

### Cell culture

The HL60 leukemia and HEK293 cell lines was purchased from the American Type Culture Collection (Manassas, VA, USA) and cultured with RPMI-1640 medium (Hyclone, Thermo Scientific, Logan City, UT, USA) supplemented with 10% fetal bovine serum (FBS; Gibco, Carlsbad, CA, USA) at 37°C in a humidified atmosphere of 5% CO_2_. The human bone marrow cells were harvested from the hips of three consenting healthy patients, two males and one female (age range, 56–68 years) following institutional review board approval from the Children’s Hospital of Chongqing Medical University (Chongqing, China). The bMSCs were isolated from the bone marrow cells via Ficoll-Paque (Amersham Pharmacia Biotech, Amersham, UK) density gradient separation medium, and the separated mononuclear cells were washed twice with phosphate-buffered saline (Beyotime Institute of Biotechnology, Shanghai, China).

### RNA extraction and quantitative polymerase chain reaction (qPCR)

Total RNAs were extracted from the bMSCs and HL60 cells via TRIzol reagent (Invitrogen Life Technologies, Carlsbad, CA, USA) according to the manufacturer’s instructions, and finally resuspended in 35 μl of preheated (68°C) nuclease-free water.

For the qPCR analysis of Wnt5a, ROR2, frizzled family receptor 5 (FZD5), β-catenin, CaMKII, cyclin D1 and β-actin, the total RNAs were transcribed to cDNAs using the PrimeScript™ RT reagent kit (Takara Bio, Inc., Shiga, Japan). The qPCR was then performed using the SYBR^®^ Green real-time PCR master mix (QPK-201; Toyobo Corporation, Osaka, Japan) and C1000™ Thermal Cycler (CXF96; Bio-Rad, Hercules, CA, USA). The Ct value of β-actin was used to normalize the mRNA levels and the primer sequences of the above genes are shown in Table I. The qPCR was performed using the following parameters: Initial hold at 95°C for 30 sec, followed by 40 cycles of 95°C for 10 sec, 60°C for 10 sec and 72°C for 20 sec.

### Construction and purification of adenovirus-mediated vectors

The adenoviruses expressing green fluorescent protein (AdGFP) and Wnt5a protein (adeno-Wnt5a) were provided by Dr T.C. He (Molecular Oncology Laboratory, Department of Surgery, The University of Chicago Medical Center, Chicago, IL, USA). The adenoviruses were propagated and purified as described prevoiously (12). Briefly, the adenoviruses were propagated into the HEK293 cells, and subsequently the successful viral infection was confirmed by GFP expression. Cell pellets were resuspended in 1× PBS and lysed using four freeze-thaw-vortex cycles. The vectors were dialyzed in a storage buffer (Promega, Madison, WI, USA) following purification using a cesium chloride gradient (Sigma-Aldrich, Shanghai, China), followed by transfection into HEK293 cells via lipofectamine 2000 (Invitrogen Life Technologies) for 24 h. Subsequently the titers of the adenovirus were determined.

### Cell proliferation assay

The cells were seeded at a density of 1×10^4^ cells per well in 96-well plates. Next, 100 μl of the culture supernatants of bMSCs, bMSCs infected with Wnt5a-encoding adenovirus (adeno-Wnt5a bMSCs) or bMSCs infected with empty adenoviral vector (adeno-vector bMSCs) (provided by T.C. He) and 100 μl of RPMI 1640 medium supplemented with 10% FBS (Sigma-Aldrich, St. Louis, MO, USA) were mixed and added to the each experimental well of the 96-well plates (12,13). Following culture for 24 or 48 h, the cell proliferation was detected using the Cell Counting Kit-8 assay (CCK-8; Beyotime Institute of Biotechnology, Haimen, China) according to the manufacturer’s instructions.

### ELISA

The protein levels of Wnt5a were measured using a human Wnt5a ELISA kit (Groundwork Biotechnology Diagnosticate Ltd., San Diego, CA, USA) according to the manufacturer’s instructions, and the absorbance was read at 450 nm using a microplate reader (SpectraMax 190, Molecular Devices LLC, Sunnyvale, CA, USA).

### Immunohistochemical (IHC) staining

For the IHC staining, the HL60 cells were seeded on microscope glass plates and non-specific staining was blocked using Dako Protein Block (Dako, Carpinteria, CA, USA) according to the manufacturer’s instructions. Next, the anti-CD13, -CD14 and -CD68 (all at a dilution of 1:200) primary antibodies were diluted in Dako antibody diluent and incubated with the cell sections for 1 h at room temperature. Finally, staining was visualized using the Dako Envision kit and developed using a DAB chromogen substrate (Dako).

### Western blot analysis

The protein expression of Ror2 (105 kDa), FZD5 (65 kDa), CaMKII (50 kDa) β-catenin (86 kDa), cyclin D1 (34 kDa), Wnt5a (45 kDa), GAPDH (37 kDa) and β-actin (43 kDa) were analyzed by western blot analysis using anti-Ror2 rabbit monoclonal (ab92379; Abcam, Cambridge, UK), anti-Frizzled 5 rabbit polyclonal (ab75234; Abcam), anti-CaMKII rabbit polyclonal (sc-13082; Santa Cruz Biotechnology, Inc., Santa Cruz, CA, USA), anti-β-catenin rabbit monoclonal (ab32572; Abcam), anti-cyclin D1 rabbit polyclonal (ab95281; Abcam), anti-Wnt5a rabbit polyclonal (2392; Cell Signaling Technology, Inc., Danvers, MA, USA), anti-GAPDH rabbit monoclonal (5174; Cell Signaling Technology, Inc.) and anti-β-actin rabbit monoclonal (4970; Cell Signaling Technology, Inc.) antibodies according to the manufacturer’s instructions.

### Statistical analysis

Data are presented as the mean ± standard deviation. Student’s t-test was used for comparisons between the two groups. P<0.05 was considered to indicate a statistically significant difference. All experiments were conducted in duplicate and the statistical analyses were performed using SPSS 17.0 software (SPSS, Inc., Chicago, IL, USA).

## Results

### Wnt5a overexpression in bMSCs induced by an adenoviral vector

**Figure 1:**
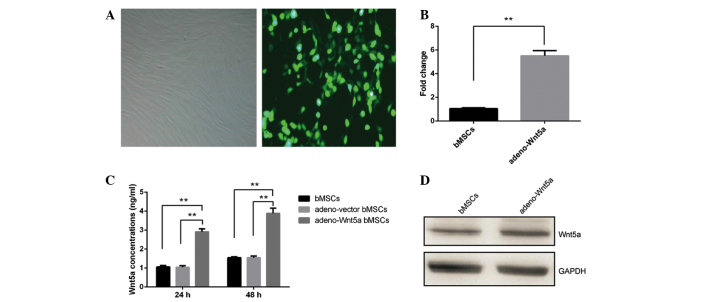
Wnt5a expression induced in bMSCs via an adenoviral vector. (A) The adeno-Wnt5a bMSCs expressed green fluorescent protein. (B and C) The mRNA and protein expression of Wnt5a in the bMSCs and adeno-Wnt5a bMSCs. (D) The protein level of Wnt5a in the culture supernatants obtained from the bMSCs, adeno-vector bMSCs and adeno-Wnt5a bMSCs. ^**^P<0.01. bMSCs, bone marrow mesenchymal cells.

To identify the role of Wnt5a derived from bMSCs in influencing the growth of HL60 cells, an adenoviral vector, adeno-Wnt5a-GFP, was constructed which expresses the Wnt5a protein in bMSCs. The bMSCs expressing GFP were sorted from non-expressing bMSCs and bMSCs infected with the adenoviruses containing adeno-Wnt5a-GFP or adeno-vector-GFP. As shown in Fig. 1A, approximately all of the bMSCs expressed GFP. The mRNA and protein levels of Wnt5a were significantly increased in the adeno-Wnt5a bMSCs compared with those in the adeno-vector bMSCs (P<0.01; Figs. 1B and C). The protein levels of Wnt5a in the culture supernatant of adeno-Wnt5a bMSCs were also significantly elevated when compared with those in the culture supernatant of adeno-vector bMSCs, following culture for 24 or 48 h (P<0.01; Fig. 1D). These results indicated that Wnt5a expression can be induced in bMSCs and secreted in the culture supernatant of the bMSCs.

### bMSC-derived Wnt5a inhibits HL60 cell proliferation

**Figure 2:**
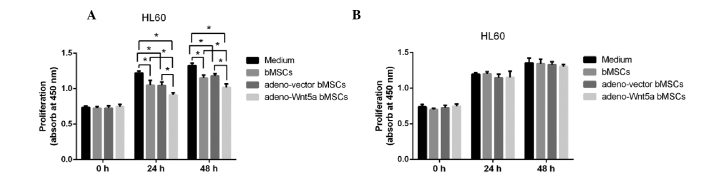
bMSC-derived Wnt5a inhibits the proliferation of HL60 cells. (A) The absorbance at 450 nm was measured following the culture of HL60 cells with the conditional medium and the culture supernatants obtained from bMSCs, adeno-vector BMSCs and adeno-Wnt5a BMSCs for 0, 24 or 48 h. (B) The absorbance at 450 nm was measured following the culture of HL60 cells with the conditional medium and the culture supernatants containing Wnt5a neutralization antibodies against bMSCs, adeno-vector bMSCs and adeno-Wnt5a bMSCs. ^*^P<0.05 and ^**^P<0.01. bMSCs, bone marrow mesenchymal cells.

To analyze whether bMSC-derived Wnt5a influences HL60 cell proliferation, the HL60 cells were stimulated with the culture supernatants of bMSCs, adeno-Wnt5a bMSCs and adeno-vector bMSCs for 24 or 48 h. The culture supernatants were collected following cell culture for 24 h and the proliferation of the HL60 cells was detected using the CCK-8 assay. As shown in Fig. 2A, the proliferation of HL60 cells was significantly decreased following stimulation with the culture supernatant of adeno-Wnt5a bMSCs when compared with that following stimulation with the culture supernatants of bMSCs and adeno-vector bMSCs (P<0.05). Although, the proliferation of HL60 cells was significantly decreased in the bMSC and adeno-vector bMSC groups when compared with that in the conditional medium of HL60 cells (P<0.05), no significant difference was identified in the proliferation of HL60 cells between the bMSC and adeno-vector bMSC groups.

To identify the role of bMSC-derived Wnt5a in the proliferation of leukemia HL60 cells, a neutralization antibody against Wnt5a was added to the culture supernatant prior to stimulation for 1 h. The proliferation of HL60 cells following stimulation with the Wnt5a-blocked culture supernatant was then detected, and no significant difference was identified in the proliferation of HL60 cells among all of the groups (Fig. 2B). These results suggested that bMSC-derived Wnt5a inhibits the proliferation of leukemia HL60 cells.

### bMSC-derived Wnt5a promotes HL60 cell maturation

**Figure 3:**
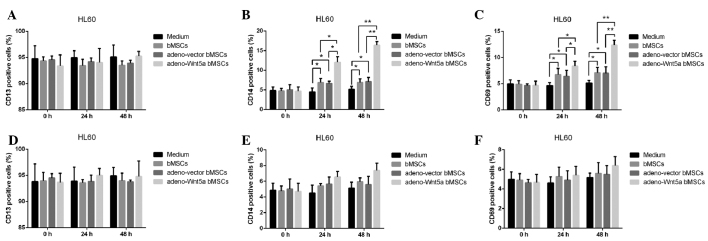
bMSC-derived Wnt5a promotes the maturation of HL60 cells. (A–C) The expression of CD13, CD14 and CD68 was evaluated by immunohistochemical staining assay following the culture of HL60 cells with conditional medium and the culture supernatants of bMSCs, adeno-vector bMSCs and adeno-Wnt5a bMSCs for 0, 24 and 48 h. (D–F) Immunohistochemical staining for CD13, CD14 and CD68 following the culture of HL60 cells with conditional medium and the culture supernatants containing Wnt5a neutralization antibodies against bMSCs, adeno-vector BMSCs and adeno-Wnt5a BMSCs for 0, 24 or 48 h. ^*^P<0.05 and ^**^P<0.01. bMSCs. bMSCs, bone marrow mesenchymal cells.

To analyze the effect of bMSC-derived Wnt5a on the maturation of HL60 cells, the culture supernatants of bMSCs, adeno-Wnt5a bMSCs and adeno-vector bMSCs were assembled following cell culture for 24 h and used to stimulate HL60 cells for 24 or 48 h. The maturation of HL60 cells was then analyzed by detecting the number of cells expressing CD13, CD14 or CD68. As shown in Fig. 3A–C, no significant difference was identified in the number of CD13-positive cells among the conditional medium, bMSC, adeno-vector bMSC and adeno-Wnt5a bMSC groups. However, a significantly increased number of CD14- and CD68-positive cells were observed in the adeno-Wnt5a bMSC group than that in the bMSC and adeno-vector bMSC groups at 24 h (P<0.05) or 48 h (P<0.01). Furthermore, a significantly increased number of CD14- and CD68-positive cells were identified in the bMSC and adeno-vector bMSC groups than that in the conditional medium group (P<0.05). However, no significant difference in the number of CD14- and CD68-positive cells was identified between the bMSC and adeno-vector bMSC groups.

To investigate the effect of bMSC-derived Wnt5a on HL60 cell maturation, the neutralization antibody against Wnt5a was added to the culture supernatants of the bMSCs, adeno-Wnt5a bMSCs or adeno-vector bMSCs for 1 h prior to stimulation. Subsequently, the number of CD13-, CD14- and CD68-positive cells was calculated and, as shown in Fig. 3D–F, no significant difference was identified in the number of positive cells among the four groups. These results indicated that bMSC-derived Wnt5a promotes the maturation of leukemia HL60 cells.

### bMSC-derived Wnt5a performs its functions by enhancing the non-canonical and inhibiting the canonical Wnt signaling pathways in HL60 cells

**Figure 4:**
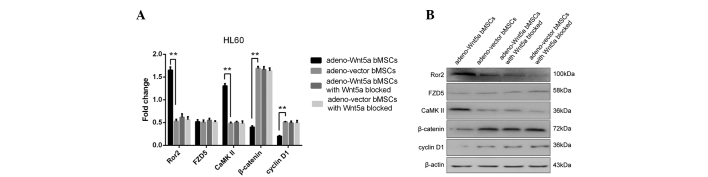
bMSC-derived Wnt5a enhances the non-canonical and inhibits the canonical Wnt signaling pathways. (A and B) The mRNA and protein expression of Ror2, FZD5, CaMKII, β-catenin, cyclin D1 and β-actin were detected via western blot analysis when the HL60 cells had been stimulated with the culture supernatants obtained from adeno-Wnt5a bMSCs or adeno-vector bMSCs, as well as the culture supernatant with blocked expression for Wnt5a. ^**^P<0.01. Ror2, receptor tyrosine kinase-like orphan receptor 2; FZD5, frizzled family receptor 5; CaMKII, Ca^2+^/calmodulin-dependent protein kinase.

It is well known that Wnt5a affects the canonical and non-canonical Wnt signaling pathways (7,14–16). However, it remains unknown whether bMSC-induced Wnt5a influences the canonical, non-canonical or the two Wnt signaling pathways. Thus, the expression of Wnt5a receptors, FZD5 and ROR2, were detected, as well as the downstream proteins, β-catenin and cyclin D1, the alteration of which is hypothesized to activate or inhibit the canonical Wnt signaling (17). In addition, the expression of CaMKII was investigated, as it is considered to alter the non-canonical Wnt signaling pathway (18). The expression of the abovementioned proteins was analyzed in HL60 cells stimulated for 48 h with the culture supernatants of adeno-Wnt5a bMSCs or adeno-vector bMSCs, or with the culture supernatant with blocked Wnt5a expression. As shown in Fig. 4, the significantly increased expression of Ror2 and CaMKII and decreased expression of β-catenin and cyclin D1 were observed in the group of cells stimulated with the culture supernatant of adeno-Wnt5a bMSCs when compared with the group stimulated with the supernatant of adeno-vector bMSCs. However, no significant difference was identified in the expression of the four proteins between the two groups stimulated with the culture supernatants of adeno-Wnt5a bMSCs or adeno-vector bMSCs containing a neutralization antibody against Wnt5a. Furthermore, no significant difference was identified in the expression of FZD5 among the four groups. These results indicated that bMSC-derived Wnt5a activates the non-canonical and inhibits the canonical Wnt signaling pathways.

## Discussion

The present study confirmed that bMSC-derived Wnt5a inhibits the proliferation of HL60 cells and promotes their maturation. Furthermore, it was identified that bMSC-derived Wnt5a activates the non-canonical and inhibits the canonical Wnt signaling pathways in HL60 cells.

Although Wnts are secreted proteins, it is difficult to purify active Wnt molecules, including Wnt5a. Therefore, the present study constructed an adeno-Wnt5a-GFP vector which expresses the Wnt5a protein in bMSCs to simulate the active Wnt5a molecule. Accumulating evidence has indicated that the Wnt5a/Ror2 pathway is associated with the differentiation fate of bMSCs (19), and that the canonical and non-canonical Wnt signaling pathways differentially affect the developmental potential of bMSCs (20). An additional study has demonstrated that bMSCs control HSC migration and proliferation via secreted molecules, including chemokine stromal derived factor-1 and Wnt5a (21). The results of our previous study indicated that Wnt5a-overexpressing bMSCs modify the proliferation and maturation of HL60 cells when the two cells are cocultured for three, five or seven days (9). Therefore, the present study focused on bMSC-derived Wnt5a to investigate its effect on the growth of leukemia HL60 cells and to determine which factor or factors alter the proliferation and maturation of HL60 cells, and whether Wnt5a or other factors are altered as a result of the overexpression of Wnt5a in bMSCs. As predicted, the culture supernatants of bMSCs, adeno-Wnt5a bMSCs and adeno-vector bMSCs were also found to suppress the proliferation of HL60 cells and promote HL60 cell maturation. In particular, the culture supernatant of adeno-Wnt5a bMSCs exhibited the most significant effect. In addition, the phenotype of the Wnt5a protein was lost when the cells were stimulated with the culture supernatant containing a neutralization antibody against Wnt5a. These results provide strong evidence that bMSC-derived Wnt5a modifies the proliferation and maturation of leukemia HL60 cells.

It is well known that Wnt5a functions in mammalian cells via binding to its receptors, including FZD5 (a common receptor) and Ror2 (a coreceptor). FZD5 belongs to the mammalian frizzled family, which mediates Wnt5a signaling via FZD3, FZD4, FZD5 and FZD8, but not FZD6, according to the assessment of the phosphorylation of disheveled protein (Dvl1 human homolog) in Drosophila Schneider 2 cells (22). In addition, mutation in the KTxxxW motif or the first or third loop of the human FZD5 has been found to prevent the binding of Dvl and the resultant signaling (23); therefore, FZD5 is crucial in Wnt5a signaling. Ror-2, however, is a single-pass transmembrane receptor with a tyrosine kinase domain, which may be more closely involved in the specific activation of Wnt5a signaling and activation of the non-canonical Wnt signaling pathway. Unlike Wnt1 and Wnt3a which activate the β-catenin pathway, Wnt5a has been frequently shown to activate the β-catenin-independent and non-canonical Wnt signaling pathways via binding to its receptors, frizzled and Ror2 (24), as well as inhibiting the β-catenin pathway (25). In contrast to the inhibitory effects of Wnt5a on the β-catenin pathway, several studies have reported that Wnt5a stimulates the β-catenin pathway (26). To investigate whether bMSC-derived Wnt5a is involved in the modification of HL60 cells, the present study detected the effect of Wnt5a on the canonical and non-canonical Wnt signaling pathways. As predicted, Wnt5a was observed to activate the non-canonical and inhibit the canonical Wnt signaling pathways.

In conclusion, the present study confirmed that bMSC-derived Wnt5a inhibits the proliferation of leukemia HL60 cells and promotes their maturation via activating the non-canonical and impairing the canonical Wnt signaling pathways.

## Figures and Tables

**Table I:** Primer sequences.

Gene	Primers	Product, bp
Wnt5a	F: 5′-TGTGGTTTAATGGTGCCTGA-3′ R: 5′-TTCGTCGTGCTCAAGGTATG-3′	253
ROR2	F: 5′-ATGGAACTGTGTGACGTACCC-3′ R: 5′-GCGAGGCCATCAGCTG-3′	186
FZD5	F: 5′-TGTCTGCTCTTCTCGGC-3′ R: 5′-CCGTCCAAAGATAAACTGCT-3′	142
β-catenin	F: 5′-TGGTTGCCTTGCTCAACA-3′ R: 5′-AGCTTGGGGTCCACCACT-3′	125
CaMKII	F: 5′-AAGATGTGCGACCCTGGAATG-3′ R: 5′-TGTAGGCGATGCAGGCTGAC-3′	784
Cyclin D1	F: 5′-CCCTCGGTGTCCTACTTCAAA-3′ R: 5′-CACCTCCTCCTCCTCCTCTTC-3′	726
β-actin	F: 5′-TTCCTTCCTGGGCATGGAGTCC-3′ R: 5′-TGGCGTACAGGTCTTTGCGG-3′	191

F, Forward; R, Reverse; ROR2, receptor tyrosine kinase-like orphan receptor 2; FZD5, frizzled family receptor 5; CaMKII, Ca^2+^/calmodulin-dependent protein kinase.
